# Supplementary material for: Multi‐dataset identification of innovative feature genes and molecular mechanisms in keratoconus
Source: J Cell Mol Med. 2024 Sep 19;28(18):e70079. doi: 10.1111/jcmm.70079 (PMC11412914; doi:10.1111/jcmm.70079)
Supplement: Supplementary file 2 — Material S2. [file JCMM-28-e70079-s002.zip › 7.GSEA analysis/1.ARL11/KEGG_BIOSYNTHESIS_OF_UNSATURATED_FATTY_ACIDS.html]

Details for gene set KEGG\_BIOSYNTHESIS\_OF\_UNSATURATED\_FATTY\_ACIDS[GSEA]

|  || Dataset | ARL11.group.cls#h\_versus\_l |
| Phenotype | group.cls#h\_versus\_l |
| Upregulated in class | h |
| GeneSet | KEGG\_BIOSYNTHESIS\_OF\_UNSATURATED\_FATTY\_ACIDS |
| Enrichment Score (ES) | 0.26971468 |
| Normalized Enrichment Score (NES) | 0.9375189 |
| Nominal p-value | 0.5610687 |
| FDR q-value | 1.0 |
| FWER p-Value | 1.0 |
Table: GSEA Results Summary

  

Fig 1: Enrichment plot: KEGG\_BIOSYNTHESIS\_OF\_UNSATURATED\_FATTY\_ACIDS      
 Profile of the Running ES Score & Positions of GeneSet Members on the Rank Ordered List

  

| SYMBOL | TITLE | RANK IN GENE LIST | RANK METRIC SCORE | RUNNING ES | CORE ENRICHMENT || 1 | BAAT | na | 614 | 0.452 | 0.2457 | Yes |
| 2 | ELOVL2 | na | 3918 | 0.203 | 0.2690 | Yes |
| 3 | FADS2 | na | 7752 | 0.097 | 0.2151 | Yes |
| 4 | HSD17B12 | na | 7822 | 0.096 | 0.2689 | Yes |
| 5 | ACOX1 | na | 9236 | 0.071 | 0.2697 | Yes |
| 6 | SCD5 | na | 11918 | 0.037 | 0.2140 | No |
| 7 | SCD | na | 12612 | 0.030 | 0.2117 | No |
| 8 | ELOVL6 | na | 13217 | 0.025 | 0.2089 | No |
| 9 | TECR | na | 14657 | 0.014 | 0.1754 | No |
| 10 | ACAA1 | na | 15725 | 0.006 | 0.1480 | No |
| 11 | ACOT7 | na | 16026 | 0.004 | 0.1414 | No |
| 12 | ACOX3 | na | 18292 | -0.012 | 0.0833 | No |
| 13 | HADHA | na | 19491 | -0.020 | 0.0606 | No |
| 14 | ACOT1 | na | 19766 | -0.022 | 0.0656 | No |
| 15 | PECR | na | 23312 | -0.054 | -0.0053 | No |
| 16 | FADS1 | na | 25051 | -0.077 | -0.0108 | No |
| 17 | ACOT4 | na | 26860 | -0.105 | -0.0015 | No |
| 18 | YOD1 | na | 27980 | -0.130 | 0.0421 | No |
| 19 | ELOVL5 | na | 32040 | -0.262 | 0.0774 | No |
Table: GSEA details [plain text format]

  

Fig 2: KEGG\_BIOSYNTHESIS\_OF\_UNSATURATED\_FATTY\_ACIDS      
 Blue-Pink O' Gram in the Space of the Analyzed GeneSet

  

Fig 3: KEGG\_BIOSYNTHESIS\_OF\_UNSATURATED\_FATTY\_ACIDS: Random ES distribution      
 Gene set null distribution of ES for **KEGG\_BIOSYNTHESIS\_OF\_UNSATURATED\_FATTY\_ACIDS**
